# Increased frequency of visits improves the efficiency of surgical global health initiatives

**DOI:** 10.1186/1916-0216-42-41

**Published:** 2013-06-20

**Authors:** Richard Byaruhanga, Ryan Rourke, Michael Awubwa, Brian D Westerberg, J Thomas Rolland, Jean-Philippe Vaccani

**Affiliations:** 1 Department of ENT, Makerere University, Kampala, Uganda; 2 St. Paul’s Rotary Hearing Clinic, Division of Otolaryngology, University of British Columbia, British Columbia, Canada; 3 Department of Otolaryngology, NYU School of Medicine, New York City, USA; 4 Department of Otolaryngology, University of Ottawa, Ottawa, Canada

## Abstract

**Background:**

The Uganda Hearing Project is a non-profit program assisting with teaching of ear surgery in Uganda. The project started with cadaveric temporal bone courses in 2003 and 2005, including donation of operating microscopes and ear instruments. In 2006, three surgical groups started regular surgical teaching visits.

**Methods:**

A retrospective chart review of all cases of middle ear surgery performed in Uganda from 2003 to 2009. Surgeries by local surgeons without foreign presence were coded as ‘local’ and those performed with assistance of visiting surgeons were coded as ‘visitors’.

**Results:**

In 2005, two middle ear surgeries using the operating microscope were done in the Ugandan teaching hospitals by Ugandan Otolaryngologists alone. From the onset of surgical visits in 2006, a total of 193 middle ear surgeries were performed - 115 tympanomastoidectomies, 77 tympanoplasties, and 1 cochlear implant. In 2006 (one surgical teaching visit), 6 middle ear surgeries were performed with visiting surgeon presence and 2 surgeries were performed by the local team alone. This increased in 2007 (2 visits) and again in 2008 (3 visits) to 34 cases with visiting surgeon presence and 48 local cases.

**Conclusions:**

The temporal bone courses and donation of operating microscopes to Ugandan hospitals have revolutionized middle ear surgery in Uganda. The surgical visits by the Uganda Hearing Project have led to a 24-fold increase in annual middle ear surgeries performed with the operating microscope by Ugandan Otolaryngologists. Increased frequency of surgical visits was correlated with an increase in local surgical output, hopefully resulting in improved care for Ugandans with ear disorders.

## Introduction

Chronic illnesses currently account for over 50% of the global burden of disease [1]. Chronic otitis media (COM) accounts for 28 000 deaths and over 2 million disability-adjusted life-years (DALYs) annually with 90% of the overall burden borne by countries in Africa, Southeast Asia and the Pacific Islands [2]. The morbidity and mortality associated with COM, through complications such as hearing loss, facial nerve paralysis, intracranial infection and sepsis, can be prevented with appropriate and timely intervention.

The number of non-governmental organizations (NGO) offering surgical care in low and middle-income countries (LMIC) has increased dramatically over the last several years [3]. However, few have studied the effect of NGO provision of these services for patients. Measurement of the effect of provision of these surgical services and sharing of lessons learned could be informative in improving efficiency of future endeavors.

The Uganda Hearing Healthcare Project is a collaborative effort between three Otolaryngology Departments of North American medical schools: the University of British Columbia (UBC), New York University (NYU) School of Medicine and the University of Ottawa (U of O). The program’s goal is to teach otologic surgery to local Ugandan Otolaryngologists in order to facilitate their evolution into independent middle ear and temporal bone surgeons using up-to-date ear instruments including microsurgical techniques and mastoid drills. Since its inception in 2000, training has focused on medical and surgical aspects of the treatment of patients with ear diseases. Early in the course of the Project, visits occurred relatively infrequently. As further equipment became available and more surgeons became aware of the project, the frequency of the visits rapidly increased.

The objective of this study was to assess the effect of the frequency of visits to Uganda by North American Otolaryngologists on the surgical output (number of patients undergoing ear surgery) during foreign surgeons’ visits and by local surgeons in the absence of a visiting Otolaryngologist.

## Methods

A retrospective chart review of all patients undergoing middle ear surgery at the participating sites in Uganda was undertaken for the study period from January 2003 to December 2009.

Procedures were classified as “middle ear surgery” if they encompassed any of the following: tympanoplasty (without mastoidectomy), tympanomastoidectomy, cochlear implantation or ossicular reconstruction. Participating sites included Mulago Hospital (the tertiary referral hospital for Uganda) and the International Hospital Kampala (IHK; a private hospital) both in Kampala; Mbale Regional Hospital; Jinja Regional Referral Hospital; and Arua Regional Hospital. These are all sites in Uganda with practicing Otolaryngologists who participated in the Uganda Hearing Healthcare Project.

For the purpose of data analysis, procedures performed by the local Ugandan Otolaryngologists without a visiting surgeon’s presence were coded as ‘Local’, and those performed in the presence of a visiting Otolaryngologist usually assisting the local Ugandan surgeons were coded as ‘Visitors’.

## Results

Institutional Ethics Board approval was obtained from Makerere Medical School, Kampala, Uganda prior to initiating this study.

### Frequency of GHI visits

During preliminary visits in 2000 and 2001, a need was identified for improvement in Uganda in provision of surgical services in otologic diseases. A cross-sectional survey of hearing impairment and ear disease was originally performed in 2001 following which two cadaveric temporal bone courses for all Ugandan Otolaryngologists and Otolaryngology Residents were organized in 2003 and 2005 (Table 1). Each course included didactic lectures and practical, cadaveric dissection teaching the principles of common ear procedures, including tympanoplasty, tympanomastoidectomy and ossicular reconstruction. The principles behind stapedotomy and cochlear implantation were taught in didactic lectures but practical aspects were not addressed in the temporal bone lab as performing these procedures in Uganda at the time was felt to be not feasible. The operating microscope was not used in Uganda prior to these courses. The courses also introduced Ugandan surgeons to mastoid drills and microsurgical ear instruments, to replace the hammer and gouge for mastoidectomy.

**Table 1:** Frequency of visits to Uganda by members of the Ugandan Hearing Health Care Team

**DATE**	**Visiting**	**Purpose:**
**2000**	Preliminary meeting with local Ugandan Otolaryngologists	Determine if a need existed for improving care of patients with ear disorders
**2001**	Formal Needs Assessment	Formal survey of prevalence of ear disorders in Masindi (a District in Western Uganda) [5]
**2003**	Temporal Bone Course; Clinical Officers Course	Introduction of Ugandan surgeons to the operating microscope; Course for Clinical Officers on medical management of common ear disorders
**2005**	Temporal Bone Course	Extension of training using the operating microscope in a laboratory setting;
**2006**	Team from NYU	Surgical visit
**2007**	Team from UBC	Surgical visit
	Team from NYU	Surgical visit
**2008**	Combined team from UBC/ U of Ottawa	Combined surgical visit
	Team from NYU	Surgical visit
**2009**	Team from U of Ottawa	Surgical visit
	Team from NYU	Surgical visit
	Team from UBC	Surgical visit

In 2006, surgical visits started occurring in which surgeries were performed cooperatively by foreign and Ugandan Otolaryngologists. In subsequent years, the number of surgical visits slowly increased as more North American Otolaryngologists became involved in the program (Table 1). As their abilities and comfort levels increased, Ugandan surgeons became primary surgeons with and later without the supervision of non-Ugandan surgeons.

### Surgical output

**Figure 1:**
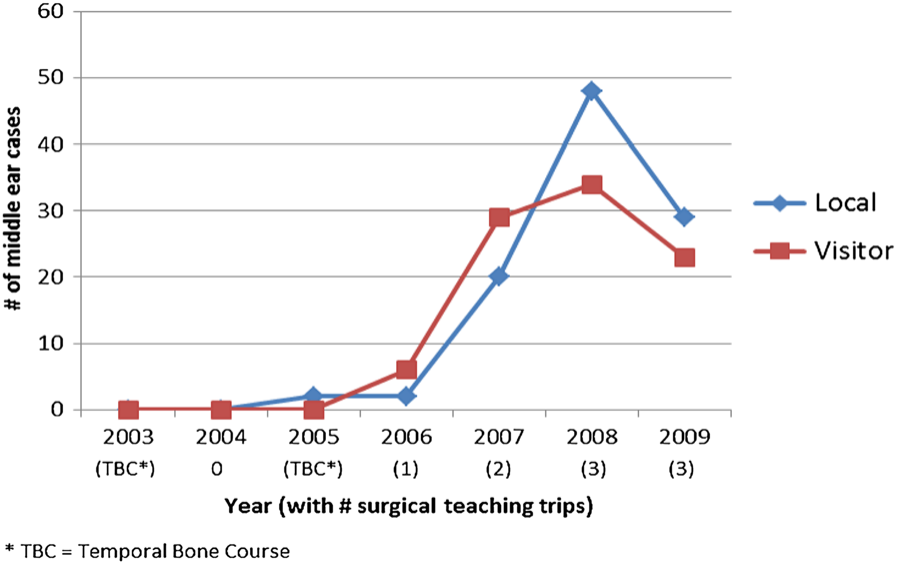
**Number of middle ear surgeries performed per year by Ugandan (“local”) and in conjunction with non-Ugandan (“visitor”) Otolaryngologists.** 2008 had a combined trip from UBC and U of O, with 3 North American Otolaryngologists.

The total number of middle ear surgical cases by both non-Ugandan and Ugandan Otolaryngologists increased over the years as the number of surgical visits by North American Otolaryngologists increased (Figure 1). In 2005, 2 middle ear surgeries using the operating microscope were done in the Ugandan teaching hospitals by Ugandan Otolaryngologists alone. These were performed after the temporal bone courses, but before the onset of surgical teaching trips. Local output stayed the same in 2006 at 2 middle ear cases, then increased to 20 cases in 2007 performed by local Otolaryngologists alone. Similarly, the surgical cases with visiting Otolaryngologist presence increased in frequency from 6 cases in 2006 to 29 cases in 2007. In 2008, there were 34 middle ear cases performed with visiting surgeon presence and 48 cases performed alone by Ugandan Otolaryngologists.

The total cases decreased in 2009 to 29 (local) and 23 (visitor) middle ear cases. This decrease occurred when the Ugandan Otolaryngologist in Arua shifted to an administrative role and no longer provided surgical services in the community. The operating microscope stayed at this hospital unused. In 2009 the Ugandan Otolaryngologist population consisted of approximately 12 surgeons; the change of career path by one surgeon in effect represented a loss of almost 10% of the national otolaryngology workforce. Also contributory, the microscope at IHK was unusable for several months until a blown microscope bulb was replaced at a subsequent visit. This center tended to be the most productive site of middle ear surgery by local Ugandan Otolaryngologists.

Overall, there were 193 middle ear surgical cases performed in Uganda between 2003 and 2009–115 tympanomastoidectomies, 77 tympanoplasties, and 1 cochlear implant. The sole cochlear implant was performed by the visiting Neurotologist from NYU with a donated implant.

## Discussion

Ugandan Otolaryngologists were conferred training in modern otologic surgical techniques through a surgical global health initiative. The effect of this initiative was to increase the surgical case volume performed by Ugandan Otolaryngologists in correlation with an increase in frequency of surgical visits by non-Ugandan Otolaryngologists. This study represents an initial step in a critical review of the Uganda Hearing Healthcare Program, a project that since its first teaching visit in 2003 has operated with the goal to create Ugandan Otolaryngologists self-sufficient in otologic surgery.

Prior to the program, most ear pathology in Uganda had been treated using hammer and gouge mastoidectomy without the operating room microscope and this technique continues to be the mainstay of surgical treatment for mastoiditis with cholesteatoma in many LMIC [4-7]. The technique does create a safe ear for many patients but often results in persistent drainage from the ear, as well as poor hearing leading to significant disability. This is in vast contrast to the progress made in the treatment of these conditions in high-income countries with the introduction of the operating microscope in the 1950s [8,9]. The introduction of modern otologic operative techniques to Ugandan surgeons will presumably improve the care of Ugandan patients with infectious ear disease and ultimately decrease the disability associated with COM, and the increase in surgical volumes is hopefully the first surrogate measure of this effect.

The increase in middle ear surgeries performed by local Otolaryngologists between 2005 and 2008 is hopefully a surrogate marker for the local surgeons having become more competent and comfortable with the operating microscope and mastoid drill. This increase in surgeries performed by local Otolaryngologists correlated with increased surgical visits by non-Ugandan Otolaryngologists and would be evidence supportive of long-term goals of global surgical initiatives, in contradistinction to one-time visits and/or “medical tourism”.

There was a demonstrated decrease of local surgical output in 2009 probably related to loss of one of 12 Ugandan Otolaryngologists to an administrative role and a non-functioning operative microscope, highlighting two common impediments in delivery of healthcare in LMIC, loss of talented surgeons to other perhaps more lucrative positions and a lack of support from biomedical engineering personnel. Admittedly, the scope of one team visit that year changed from previous surgical visits to focus more on general otolaryngological skills and thyroid surgery, resulting also in a decline in the total number of otologic cases done by the visiting team in 2009.

The Uganda Hearing Healthcare program has worked at a local level with Ugandan Otolaryngologists, an approach that seems to be crucial to make a project both successful and sustainable in LMIC [10-12]. Other ramifications for Uganda and the field of Otolaryngology of the increased productivity are immeasurable. Although this study looks at the output over just a few years, it is hoped the skills acquired by Ugandan surgeons will not only allow the provision of safe and effective middle ear surgery to their patients, but will also allow them to teach these new techniques to the next generation of Ugandan Otolaryngologists. Furthermore, in general there has been a lack of interest by medical school graduates in LMIC for surgical disciplines^13^. In Uganda, a large proportion of healthcare is funded by international donors and non-Governmental Organizations with an interest in provision of services for non-surgical conditions such as population health and infectious disease [13,14]. Surgical specialties have been underfunded and undervalued. Exposing medical students to the scope of services possible in surgical specialties through provision of donated surgical equipment may influence them to pursue further training in otolaryngology-head and neck surgery. Such a long-term effect will be hard to measure in any impact analysis. However, the number of Otolaryngology-Head and Neck Surgery Residents admitted to training positions in Makerere University has steadily increased as well over the course of the program.

An assessment of the quality of education given during the surgical trips is challenging to perform due to the lack of a hospital-based patient charting system. Follow-up of patients is difficult, given distances traveled by patients and the financial implications of this travel. However, the success of the Program would be better measured with an assessment of the success for instance in closure of tympanic membrane perforation following tympanoplasty surgery. To date, we have been unable to obtain these data, although an attempt is being made to address this deficiency as part of the program. The issue of barriers to access to care is not easily surmountable given the diversity of contributory factors (Mick et al., unpublished data).

The Uganda Hearing Healthcare Project is now expanding to include other specialties within Otolaryngology, such as pediatric otolaryngology, head and neck surgery, laryngology, rhinology and other general otolaryngology surgery. This expansion is occurring at the request of Ugandan Otolaryngologists with the hope of providing similar sustainable teaching for these areas of Otolaryngology-Head and Neck Surgery. The project has also included provision of services in related disciplines of Anesthesiology, Audiology, Speech Language Pathology, Nursing and Biomedical Engineering, disciplines essential to provision of quality services in otologic surgery.

## Conclusions

The Uganda Hearing Healthcare Project is a non-profit program that began in 2001 with the objective of teaching otologic micro-surgical techniques to Ugandan Otolaryngologists. Temporal bone courses in 2003 and 2005, introduction of the operating microscopes and mastoid drills, and subsequent increased frequency of surgical teaching visits have led to a 24 fold increase in middle ear surgical output with the operating microscope by Ugandan Otolaryngologists over just six years. Unique barriers to local surgical output were encountered during the study period, such as loss of a trained Otolaryngologist to an administrative role, and difficulty fixing specialized equipment. Such dilemmas are important learning points for this and other global health initiatives. Future teaching trips will continue to occur with hopes of ongoing education, increasing local productivity and improving patient safety in the field of Otolaryngology.

## Competing interests

The authors declare that they have no competing interests.

## Authors’ contributions

RB was involved in data collection, analysis and final approval: RR was involved in data collection, analysis, drafting of manuscript and final approval; MA was involved in conception and design and reviewing final manuscript; BW was involved in conception, data analysis and reviewing final manuscript; JTR was involved in critical revision of article and final approval; JPV was involved in conception and design, data analysis, critical revision of article and final approval. all authors read and approved the final manuscript.
